# Postinfection Metabolic Reprogramming of the Murine Trigeminal Ganglion Limits Herpes Simplex Virus-1 Replication

**DOI:** 10.1128/mbio.02194-22

**Published:** 2022-08-31

**Authors:** Chandrashekhar D. Patil, Rahul K. Suryawanshi, Divya Kapoor, Deepak Shukla

**Affiliations:** a Department of Ophthalmology and Visual Sciences, University of Illinois at Chicago, Chicago, Illinois, USA; b Department of Microbiology and Immunology, University of Illinois at Chicago, Chicago, Illinois, USA; University of North Carolina at Chapel Hill

**Keywords:** herpesvirus, latency, metabolomics, trigeminal ganglion, polyamines

## Abstract

Herpes simplex virus type-1 (HSV-1) infections are known to alter the host metabolism for efficient propagation *in vitro*. However, *in vivo* metabolic perturbations upon prolonged HSV-1 infection remain poorly understood. We used high-resolution liquid chromatography coupled with mass spectrometry (LC-MS) and functional assays to determine the state of the trigeminal ganglion (TG) tissue metabolism upon prolonged corneal HSV-1 infection in a murine model. The metabolomics data indicated significant alterations in the host metabolic profile. After HSV-1 infection, the TG microenvironment assumed downregulation of central carbon metabolism and nucleotide synthesis pathways. We validated our observations using *in vitro* and *ex vivo* models through targeted inhibition of crucial metabolic polyamine pathways identified in our metabolomics screen. Our findings collectively suggested that HSV-1 infection altered the host metabolic product regulations that limit the energy and macromolecular precursors required for viral replication.

## INTRODUCTION

Herpes simplex virus 1 (HSV-1) is a double-stranded DNA virus and a prototypic member of the alpha-herpesviruses (1). After entry and infection at the primary mucosal tissue site, the virus progresses to establish latency in the TG (2, 3). The virus frequently reactivates from latency and causes pathological lesions, manifesting as cold sores, fever blisters, keratoconjunctivitis, keratitis, and rarely encephalitis (4, 5). Like many other viruses, HSV-1 also perturbs the host metabolic homeostasis during infection for its benefit, which helps support viral growth and transmission. HSV's unique ability to encode several metabolic enzymes like thymidine kinase (TK), ribonucleotide reductase, dUTPase, and uracil DNA glycosylase (6) provides us with multiple options to target and exploit for potential therapeutics against the virus (1). The nucleoside analog acyclovir is one such compound that is clinically prescribed and effectively targets viral TK (7). How HSV-1 may be mediating the metabolic microenvironment and regulating players in specific pathways will significantly advance current knowledge while exposing new, alternate strategies to fight and curb the virus in both lytic and latent states.

Recent technological developments for measuring various metabolites present in single samples allow for in-depth analyses of virus-host metabolic interactions. The use of liquid chromatography coupled with mass spectrometry (LC-MS) has enabled the direct measurement of many extractable cellular metabolites (8). Early studies that analyzed alterations in the global metabolome during infection utilized Human cytomegalovirus (HCMV). One such study highlighted specific major HCMV-mediated alterations in host metabolic pathways that included glycolysis and fatty acid synthesis (9). Subsequent metabolic studies on acute HSV-1 infection in cultured cells have shown similar modulation of host metabolism, such as increased glycolysis and the anaplerotic influx into the tricarboxylic acid (TCA) cycle through the activity of pyruvate carboxylase and upregulation of pyrimidine nucleotides synthesis (10, 11). While these *in vitro* studies provide a foundation, there remains a need to examine *in vivo* models for metabolic profiling to overcome the current limitations associated with *in vitro* studies, such as nutrient requirement discrepancies and failure in mimicking the dynamic tissue microenvironment (12). In addition, any metabolic alterations in the TG during prolonged HSV-1 infection remain poorly understood (2, 13). The latent phase is an attractive target for better management of HSV-1 infection due to its associations with HSV-1 recurrence (14). Newer data suggest that latent viral particles may be in a dynamic process, constantly attempting to always reactivate and expressing low levels of specific viral proteins (15).

We applied a metabolite profiling approach to a murine model of HSV-1 latent infection in the current study. We further validated our observations with *in vitro* and *ex vivo* HSV-1 infection models to recapitulate the active infection scenario. We found that HSV-1 persisted in a significantly different metabolic TG tissue environment, demonstrating downregulation of central carbon metabolism and nucleotide synthesis. Changes in levels of nucleotides, amino acids, and carbohydrates represent important intrinsic host mechanisms that affected the outcome of HSV-1 infection. Our findings may have implications for the regulation of viral infections and the development of target-based therapeutics.

## RESULTS

### HSV-1 infection altered the metabolic content of murine trigeminal ganglia.

**FIG 1:**
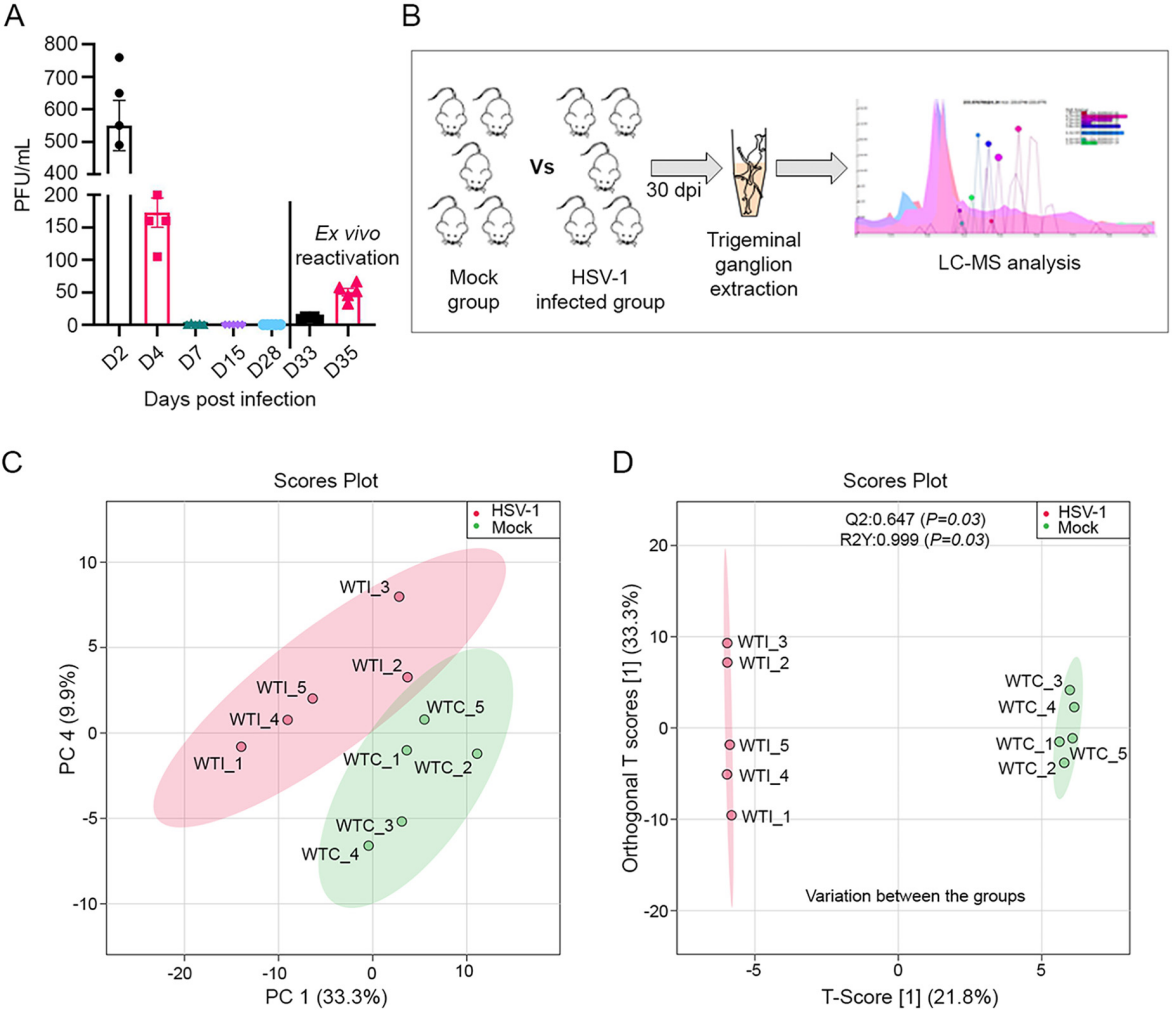
HSV-1 infection established latency in murine TGs and altered the host tissue metabolome. (A) Graph showing ocular viral shedding over time with HSV-1 McKrae infection (*n* = 5). Eyewashes were taken from the infected animals up to day 28 postinfection and on day 30 the animals were sacrificed to observe virus reactivation. TGs were collected and cultured *ex vivo*. Culture medium supernatants were collected on day 3 and day 5 after reactivation and used for plaque assays. (B) Schematic representing experimental workflow. Animals were either mock or HSV-1 infected through the ocular route with HSV-1 McKrae infection using 1 × 10^5^ PFU/mL per animal (*n* = 5). Following 30 days postinfection, TGs were collected to analyze metabolites through LC-MS/MS. (C) Graph showing principal-component analysis (PCA)-X score plot (PC-1 and PC-4) of all analyzed samples with (red) or without (green) HSV-1 infection. (D) The OPLS-DA score scatterplots for metabolic profiles of the mock-infected (green dots) and HSV-1 infected (red dots) groups showed clear discrimination between the two groups. R2Y is the variation in Y that the model defines. Q2 is the predictive ability of the model with Q2 > 0.5 indicating a good model quality. Good permutation testing was achieved if R2Y and Q2 values of the models, based on the permutated data, were significantly lower than those based on the actual data set. WTC, wild type control; WTI, wild type infected.

Before investigating the metabolic changes in HSV-1 infected mice, we confirmed the HSV-1 infection in a murine model of ocular infection. We observed a gradual decrease in viral shedding in ocular tissue through plaque assays that ended in complete loss of plaque counts from day 7 onwards up to 28 d postinfection (dpi) (Fig. 1A). Earlier reports suggest an establishment of latency after 21 to 28 days after primary infection (16). To confirm the presence of the latent virus, we extracted the TG at 30 dpi and observed the reactivation of HSV-1 *ex vivo* (Fig. 1A). Our experimental scheme was designed to study the metabolic changes in the TG-harboring latent herpesvirus (Fig. 1B). Metabolic profiling of the TG was performed at 30 dpi to determine global changes in host cell metabolism during established latency. The relative abundances of tissue lysate metabolites were analyzed using high-performance liquid chromatography-tandem mass spectrometry (HPLC-MS/MS) analysis. The identification of metabolites was based on an in-house library of analytical reference standards that accounts for the molecular ion's accurate mass (*m/z*-value), the relative retention time (concerning the internal standard), and the 13C/12C isotope ratio. After analysis, 173 distinctly characterized metabolites were identified. We excluded 22 metabolites that did not show good separation and an additional 42, which were less conclusive among the 237 total metabolites detected on our screen.

The data set was processed using principal component analysis (PCA) and an unsupervised statistical approach. The PCA analysis showed good clustering between HSV-1 infected and mock-infected animal groups (Fig. 1C), indicating good analytical reproducibility and reliability of the data set. A general overview was also established using OPLS-DA (orthogonal projections to latent structures discriminant analysis) with corresponding data sets for the metabolic effects of HSV-1 infection at 30 dpi. OPLS-DA showed clear discrimination between HSV-1 infected and mock-infected tissue samples, suggesting that the metabolome of the infected group was significantly modified due to prior HSV-1 infection (Fig. 1D). The data sets were validated by cross-validated analysis of variance (CV-ANOVA) (*P* < 0.05) and the permutation test (*n* = 100). The OPLS-DA loading plot of the HSV-1 infected, and the mock-infected group showed high ‘between-group separation while ‘with-in’ group variability was relatively conserved in the mock-infected and varied in the HSV-1 infected group. An informative graphical representation of the between groups and within-group variations is displayed in Fig. 1D.

### HSV-1 infection caused changes in key metabolites.

**FIG 2:**
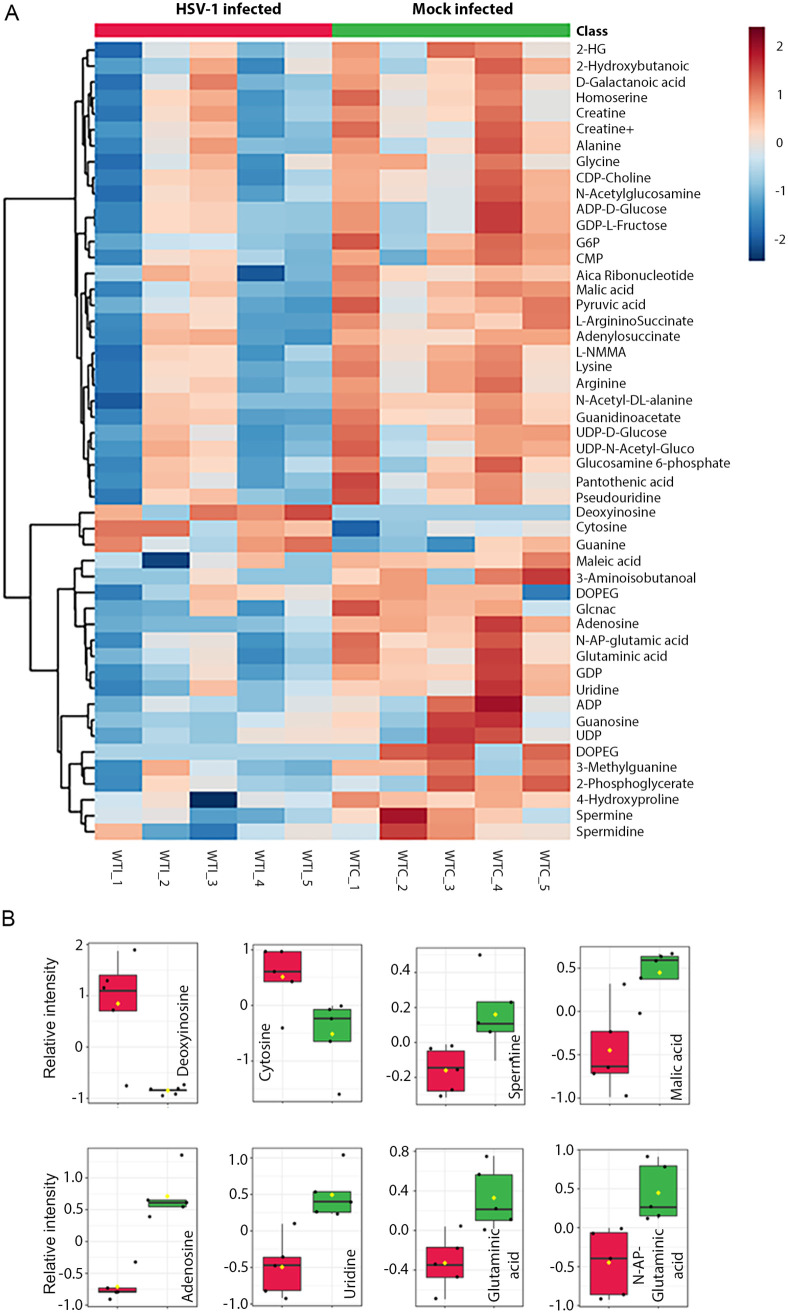
Characteristic metabolites in response to HSV-1 latency. (A) Heat map of unsupervised hierarchical cluster analysis (Ward cluster algorithm) with a dendrogram (Euclidean distance measure) for classifying top-ranked tissue metabolites (log-transformed and normalized) associated with infection. The increasing gradient of red color indicates upregulation, while an increasing gradient of blue color indicates downregulation of metabolite content. (B) Bar plots showing a comparison of relative intensities of selective metabolites observed in the TGs isolated from mice in the mock (green) and infected (red) groups (*P* < 0.05). The *y*-axis represents the relative intensity of the metabolites.

An unsupervised hierarchical cluster analysis using the Ward cluster algorithm was performed to analyze the significantly altered metabolites in mock or virus-infected samples and represented with a heatmap (Fig. 2A). The results indicated that HSV-1 infection influenced the host's metabolic profile. Only two metabolites, deoxyinosine, and cytosine were upregulated, while most of the metabolites were downregulated in the virus-infected group (Fig. 2A). Notably, spermine, the product of the polyamine pathway, was significantly downregulated in infected samples (Fig. 2B). Additionally, several purine and pyrimidine nucleobase metabolites, such as guanidine, uridine, guanosine, and glutamine, were significantly decreased in virus-infected TGs compared to mock controls. The data suggested a relative scarcity of substrates required for DNA and RNA biosynthesis in the TG. This nutrient-depleted and nonproliferating neuronal cell environment possibly limited the DNA replication capability of HSV, forcing the virus to remain latent.

### HSV-1 infection affected energy, nucleotide, and protein synthesis pathways.

**FIG 3:**
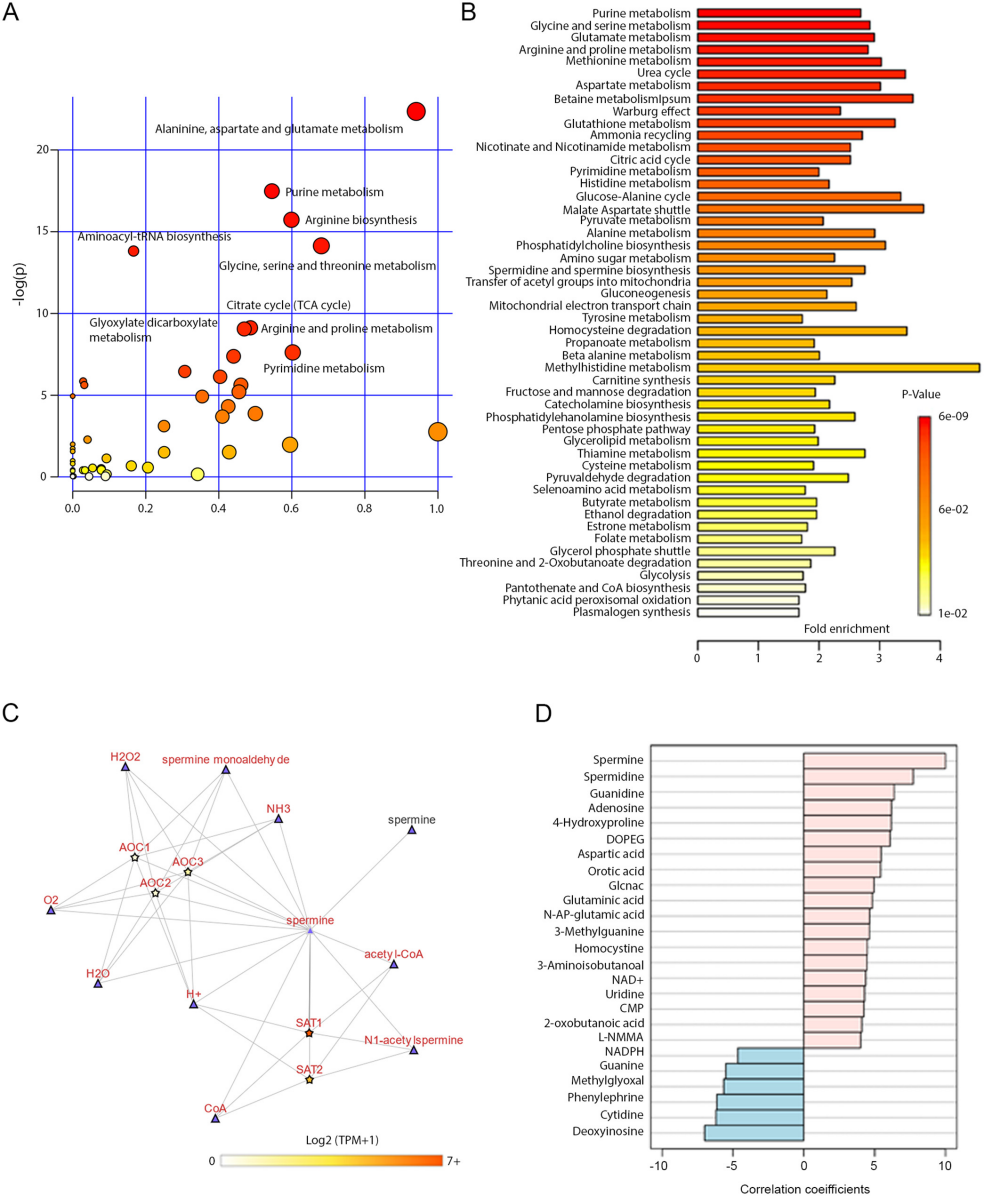
Latent HSV-1 infection affected biosynthetic pathways involving spermine biosynthesis. (A) KEGG pathway (Mus musculus (mouse)) library-based analysis of mock- or HSV-1 infected TG tissue samples. The hypergeometric test identified significantly affected pathways (red circles). (B) Enrichment analysis with KEGG pathway library of Mus musculus (mouse) showing the affected pathways. (C) Metabolic interaction partners for spermine in Arginine and proline pathway were explored using the Metabolic Atlas project (MetAtlas). A connected network graph shows the other metabolites (triangle-shaped) or genes (star-shaped) with which spermine interacts. A spermine node involved in SAT1 mediated acetyl transfer reaction (reaction id MAR06930) is highlighted while other reaction partners are grayed out. The directionality of each edge is indicated as a triangle. The RNA expression levels of interaction partners from cerebral cortex tissue are applied from the Human Protein Atlas (www.proteinatlas.org). The color of the gene’s nodes represents transcript per million (TPM). (D) The top 25 metabolites were correlated with spermine. The correlation pattern between mock versus HSV-1 infection conditions is presented. Correlation coefficient calculated by Pearson r distance measure.

Pathway analysis was performed to explore the possible transition in metabolic activity between latently infected and mock-infected samples for all identified metabolites using the MetaboAnalyst platform. The pathway impact was measured relative to all the metabolites of a single pathway for the overall spectrum of metabolites (17). An increasing impact score (0 to 1) indicated highly impacted pathways. The results from the pathway analysis are detailed in Table 1 as well as presented graphically with the metabolome view shown in Fig. 3A. Due to latent HSV-1 infection, the altered pathways are broadly relevant to amino acid synthesis, nucleotide synthesis, and energy pathways. These pathways contribute to the TCA cycle, arginine biosynthesis, arginine and proline metabolism, glycine, serine, and threonine metabolism, and aminoacyl-tRNA biosynthesis.

**TABLE 1:** Top pathways affected by latent HSV-1 infection from a pairwise metabolite pathway analysis comparing the identified metabolite contents between mock or HSV 1 infection

Pathway name	Match status^*a*^	Raw *P*^*b*^	Holm *P*^*c*^	FDR^*d*^	Impact^*e*^
Alanine, aspartate and glutamate metabolism	17/28	1.95E−10	1.64E−08	1.64E−08	0.94071
Purine metabolism	24/66	2.56E−08	2.12E−06	1.07E−06	0.54551
Arginine biosynthesis	44/118	1.48E−07	1.21E−05	4.13E−06	0.59898
Glycine, serine, and threonine metabolism	15/34	7.26E−07	5.88E−05	1.52E−05	0.68084
Aminoacyl-tRNA biosynthesis	18/48	9.98E−07	7.98E−05	1.68E−05	0.16667
Arginine and proline metabolism	13/38	0.000109	0.008629	0.001406	0.48672
Citrate cycle (TCA cycle)	9/20	0.000117	0.00914	0.001406	0.46979
Cysteine and methionine metabolism	11/33	0.000492	0.037888	0.005167	0.60257
Pyrimidine metabolism	12/39	0.000625	0.047467	0.005829	0.44053
Glyoxylate and dicarboxylate metabolism	10/32	0.001579	0.1184	0.013261	0.30689
Glutathione metabolism	9/28	0.002185	0.16166	0.016683	0.40375
Pantothenate and CoA biosynthesis	7/19	0.002882	0.21042	0.020177	0.02857
Butanoate metabolism	6/15	0.003623	0.26083	0.021735	0.03175
Nicotinate and nicotinamide metabolism	6/15	0.003623	0.26083	0.021735	0.46053
Beta-alanine metabolism	7/21	0.005485	0.38395	0.030716	0.45522
Valine, leucine, and isoleucine biosynthesis	4/8	0.007141	0.49275	0.036104	0
Pyruvate metabolism	7/22	0.007307	0.49686	0.036104	0.35458

a Total number of compounds in the pathway with the hits from the matched number from the user uploaded data.

b Original *P* value calculated from the enrichment analysis.

c *P* value adjusted by the Holm-Bonferroni method.

d *P* value adjusted using the false discovery rate.

e Pathway impact value calculated from pathway topology analysis. Pathways with the most hits, highest Holm adjusted *P* (FDR < 0.05), and highest pathway impact values are considered the most significantly affected pathways.

Further enrichment analysis with the KEGG pathway library of Mus musculus (mouse) showed notably affected pathways such as polyamine synthesis (Fig. 3B). Polyamines like spermine are commonly known to regulate both host cellular processes and viral processes during transcription and translation. Arginine and proline metabolism were also clearly affected. We examined the interaction of spermine within the arginine and proline pathway and found that spermine participates in three different reactions (Fig. 3C). One was the direct transport of spermine from the cytosol to the peroxisome. The other two reactions were independently mediated through SAT-1 and AOC1 (amine oxidase, copper containing 1).

We further analyzed the RNA expression levels of spermine interactive genes from the human cerebral cortex tissue data set (https://www.proteinatlas.org/). The SAT-1 mRNA levels are higher than AOC1, which suggested that SAT1-mediated acetylation of spermine could serve as a regulatory process in the neuronal tissue. The correlation pattern also showed the interaction of spermine with top metabolites responsible for differentiating metabolic conditions between mock and HSV-1 infected tissue samples (Fig. 3D). We focused on the spermidine and spermine biosynthesis pathway (*P* = 0.0039) for subsequent *in vitro* and *ex vivo* validation studies to establish direct relevance and further prove the concept.

### DenSpm treatment blocked HSV-1 infection *in vitro*.

**FIG 4:**
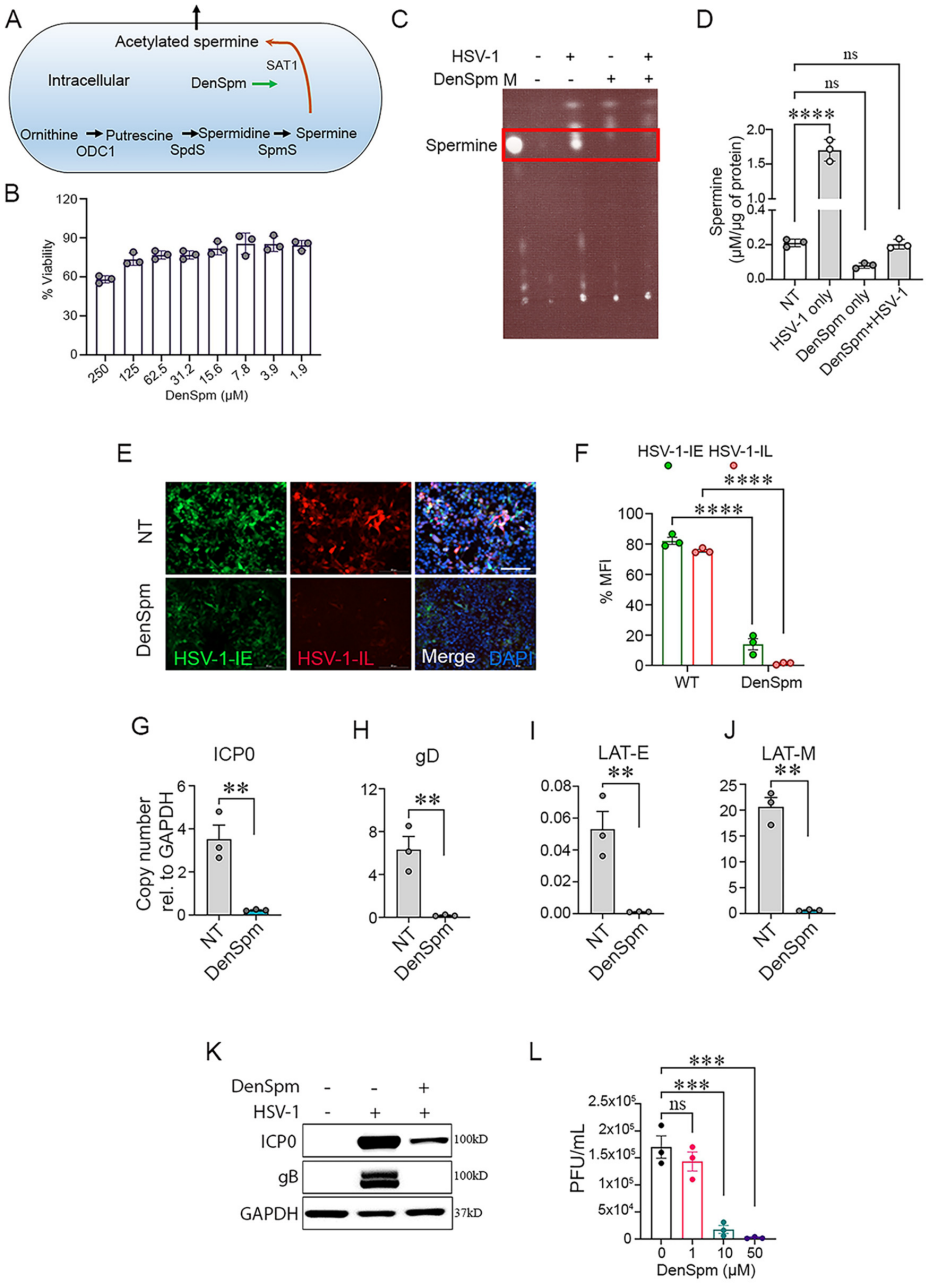
A spermine analog, DenSpm, inhibited HSV-1 infection *in vitro*. (A) Schematic representing target site for DenSpm action in the spermine synthesis pathway. (B) MTT assay showing dose-dependent viability in HCE cells after DenSpm treatment. (C) A thin-layer chromatography plate showed spermine detection in HCE cells with or without DenSpm treatment during HSV-1 or mock infected conditions. Letter M shows marker spot for standard spermine. (D) The quantitative levels of spermine in experimental conditions were measured with zone elution fluorescence assay (Ex 365, Em 505) with BioTek synergy H1 microplate reader. (E) HCE cells were pretreated with DenSpm or vehicle control, DMSO for 72 h followed by an infected with HSV-1 KOS at 0.1 MOI. At 2 hpi, the cells were again treated with DenSpm or DMSO. Representative fluorescence micrograph of HCE cells indicates HSV-1 infection. Green and red fluorescence represent the transcription of immediate early and true-late viral genes, respectively. Images were captured using Biotek Lionheart FX automated microscope under a 10× objective. Scale bar, 200 μm. (F) Quantification of fluorescence imaging using Biotek image analysis software. Graph showing expression of transcripts of (G) HSV-1 ICP-0, (H) HSV-1 gD, (I) HSV-1 latency-associated transcript enhancer (LAT-E), (J) HSV-1 latency-associated transcript major (LAT-M) as determined by RT-qPCR (K) Representative micrograph of immunoblots for HSV-1 ICP0, gB and GAPDH. (L) Graph showing generation of mature virus particles (PFU/mL) from DenSpm treated and infected HCE cells with respective concentrations (μM). Significance was determined by one-way ANOVA with Sidak multiple comparisons. Student *t* test was performed for statistical analysis (α = 0.05). *, *P* < 0.05; **, *P* < 0.01; ***, *P* < 0.001; ****, *P* < 0.0001. ns, not significant; NT, no treatment.

Given the decrease in spermine levels in the TGs of HSV-1 infected animals and its relation to the polyamine pathway, we hypothesized that spermine was essential for acute HSV-1 infection and could act as a therapeutic biomarker for active HSV-1 infection. A pharmacological analog of spermine was used to evaluate its impact on HSV-1 replication to test the hypothesis. N1,N11-diethylnorspermine (DenSpm) was a synthetic polyamine analog known to induce the expression of spermidine/spermine N1-acetyltransferase-1 (SAT-1) and thereby reduce polyamine levels in mammalian cells (Fig. 4A). *In vitro* experiments were performed with human corneal epithelial (HCE) cells due to their physiological relevance to the *in vivo* ocular infection model. We checked the viability of HCEs in different concentrations of DenSpm through an MTT (3-(4, 5-dimethylthiazolyl-2)-2, 5-diphenyltetrazolium bromide) assay. More than 85% of cells were viable at relatively high DenSpm concentrations (31.2 to 125μM) (Fig. 4B). We then checked whether the addition of DenSpm decreased spermine levels (Fig. 4C and D). Thin layer chromatography (TLC) and quantitative estimation showed significant downregulation of spermine levels in the presence of DenSpm with or without HSV-1 infection. Infection without treatment significantly increased spermine levels.

For the next set of experiments, HCE cells were first treated with DenSpm (10 μM) for 72 h and then infected with 0.1 multiplicity of infection (MOI) of an HSV-1 KOS strain, which expresses green fluorescence protein (GFP) under an immediate early viral gene expression promoter and red fluorescence protein (RFP) under an immediate-late viral gene expression promoter (Fig. 4E). At 2 h postinfection (hpi), the cells were replenished with DenSpm or DMSO. Fluorescence images taken at 24 hpi showed nearly equivalent GFP and RFP expression levels in mock-treated cells. However, DenSpm treated cells displayed reduced levels of both GFP and RFP. Quantification of the imaging data suggested that late viral gene expression might be restricted with spermine inhibition (Fig. 4F). The diminished expression of RFP was further confirmed by analyzing viral transcripts and proteins (Fig. 4G to K). Furthermore, plaque assay showed that DenSpm inhibited the production of mature virus particles in a dose-dependent manner, with no plaques observed at a 50 μM concentration (Fig. 4L).

### SAT-1 induction limited HSV-1 infection without inducing an immune response.

**FIG 5:**
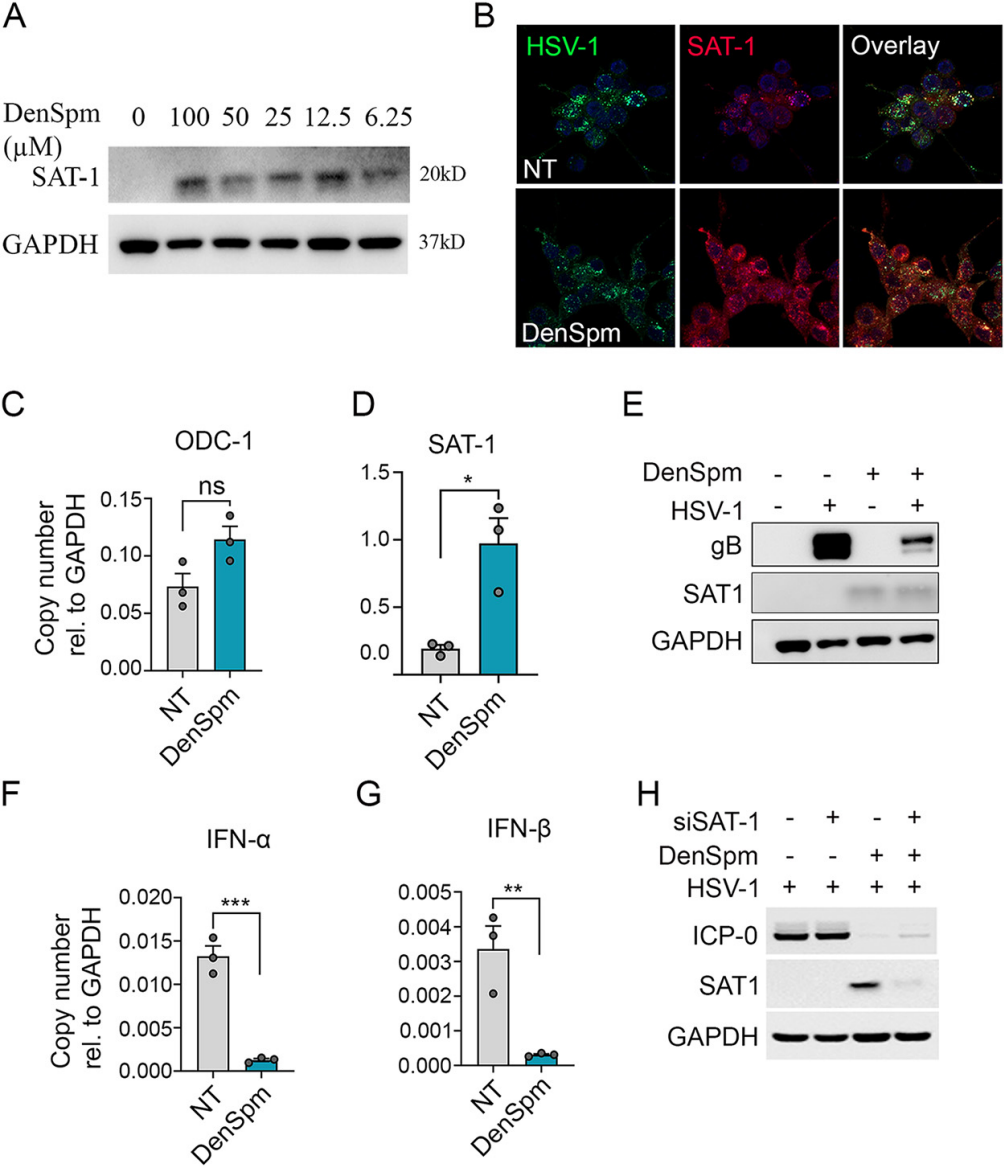
SAT-1 mediated HSV-1 regulation. (A) DenSpm induced SAT1 expression in HCE cells at all the tested concentrations. (B) Representative immunofluorescence micrograph of confocal microscopy showing expression of SAT-1 in mock or DenSpm treated and HSV-1 infected HCE cells. Micrograph representing (C) ODC-1, (D) SAT-1 transcript, and (E) protein levels of SAT-1 in the presence or absence of DenSpm treatment. Graphs showing transcript levels for (F) IFN-α and (G) IFN-β in HSV-1 infected HCE cells treated with mock or DenSpm. (H) Western blot showing silencing of SAT-1 favors the HSV-1 infection. Student *t* test was performed for statistical analysis (α = 0.05). *, *P* < 0.05; **, *P* < 0.01; ***, *P* < 0.001. ns, not significant; NT, no treatment.

Because DenSpm treatment induced the expression of SAT-1, we confirmed the induction in SAT-1 protein levels at different DenSpm concentrations (Fig. 5A). SAT-1 levels were induced independently of DenSpm concentration. This further confirmed the SAT-1 sensitivity toward the spermine analog. Expression of SAT-1 was also verified upon DenSpm treatment in HCE cells with confocal microscopy (Fig. 5B), transcript levels (Fig. 5C and D), and Western blotting (Fig. 5E). Interestingly, the confocal immunofluorescence microscopy revealed perinuclear localization of SAT-1, particularly in HSV-1 infected cells. However, DenSpm treatment did not affect the upstream components of the polyamine pathway (e.g., ODC1), as shown in Fig. 5C. A comparative quantitative real-time (qRT)-PCR analysis of HSV-1 or mock-infected HCEs either treated or untreated with DenSpm showed differences in the transcription levels of immune response genes. Cytokine (interferon (IFN)-α and IFN-β) expression was significantly upregulated in untreated HSV-1 infected cells (Fig. 5F and G). To further check if silencing of the SAT-1 gene could favor the HSV-1 infection, we transfected HCEs with a siRNA targeting SAT-1 and observed the effect on the viral infection (Fig. 5H). Successful knockdown of SAT-1 in HCEs treated with DenSpm showed a slight increase in the virus-encoded protein ICP0 compared to the control set. Our findings showed that increased SAT-1 expression inhibited the HSV-1 replication and suppressed the need for cytokine upregulation. The known function of SAT-1 in converting spermine to acetyl-spermine is likely to maintain intracellular spermine levels. However, we did not find any significant change in HSV-1 infection levels upon external addition of spermine (unpublished data).

### DenSpm treatment restricted HSV-1 infection in murine trigeminal ganglia, human neuronal cells, and human corneas.

**FIG 6:**
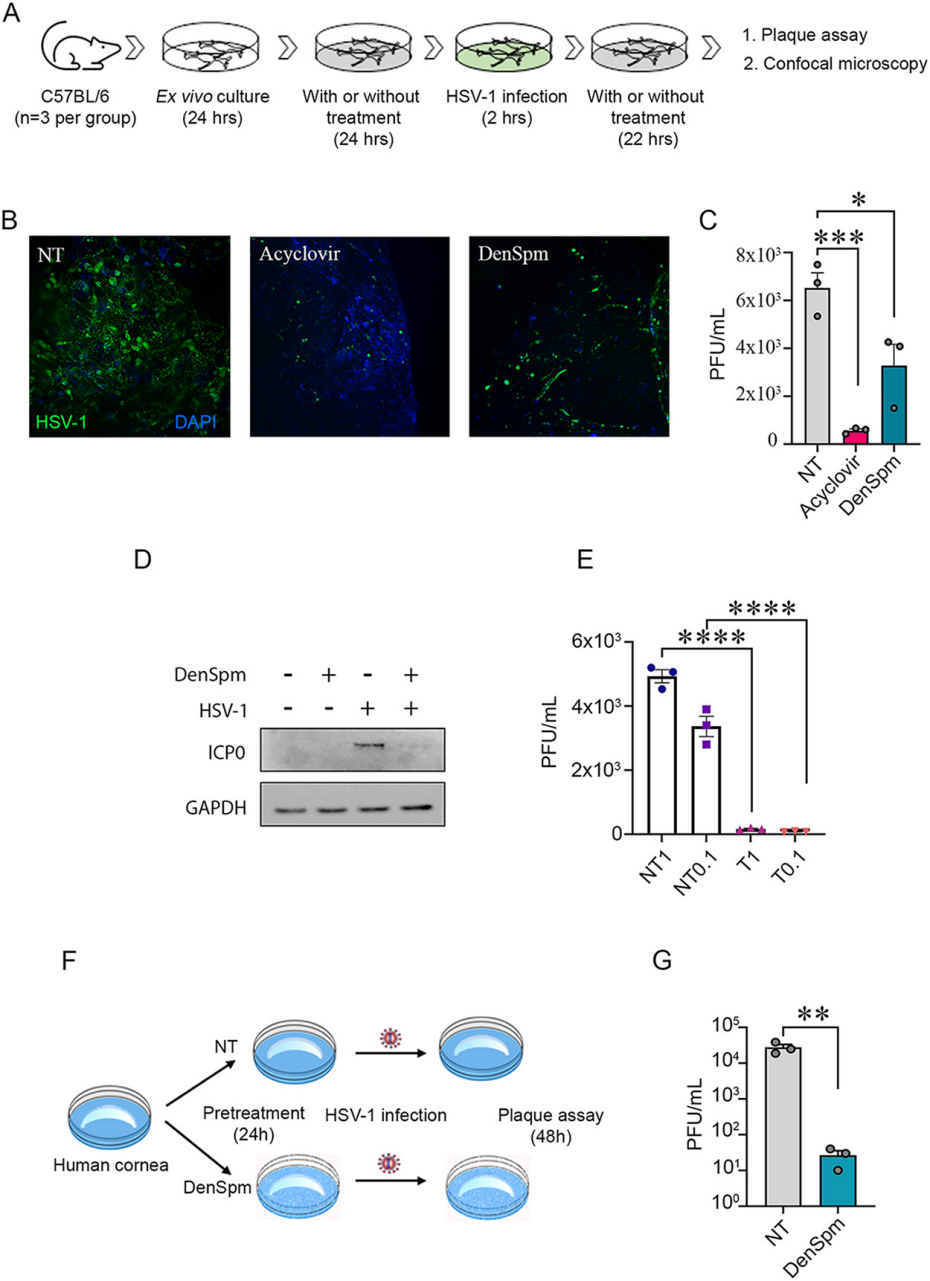
DenSpm treatment inhibited HSV-1 infection in tissue explants and neurons. (A) Schematic showing murine TG tissue collection, culture, pretreatment, and infection. (B) Representative confocal microscopy images of HSV-1 infected TGs pretreated with pharmacological inhibitors or mock. Acyclovir and DenSpm were used at 10 μM concentration for the murine tissue pretreatment. After 24 h, the tissues were infected with HSV-1 17 GFP strain (1 × 10^5^ PFU). At 2 hpi, the tissues were again added to DenSpm- or acyclovir-containing medium. (C) Graph showing generation of mature virus particles (PFU/mL) in DenSpm, acyclovir, and mock-treated murine TGs. (D) Representative Western blot for HSV-1 ICP0 protein levels in LUHMES cells (E) Graph showing viral plaque numbers at two different MOIs of HSV-1 infected and DenSpm treated LUHMES cells. T denotes treated, and NT denotes nontreated. (F) Schematic showing experimental set-up to test DenSpm efficacy on human corneal explants. (G) Graph showing antiviral efficacy in *ex vivo* cultured human corneal tissues, treated with mock or DenSpm (10 μM). Significance was determined by one-way ANOVA with Sidak multiple comparisons. Student *t* test was performed for statistical analysis (α = 0.05). *, *P* < 0.05; **, *P* < 0.01; ***, *P* < 0.001; ****, *P* < 0.0001. ns, not significant; NT, no treatment.

To add physiological relevance to *in vitro* cell culture findings, we utilized a tissue model of HSV-1 infection (18). The *ex vivo* cultured murine TGs were processed as per Fig. 6A. Confocal microscopy showed apparent differences in HSV-1 infectivity as determined by the level of GFP between drug-treated and mock-treated tissues. Tissue pretreated with acyclovir (50 μM) as positive control showed the most negligible GFP fluorescence, followed by DenSpm treated tissue (Fig. 6B). The same tissue samples were further processed to quantify the formation of mature virus particles (Fig. 6C). Significant differences in plaque assay data confirmed the reduction in HSV-1 infection using prophylactic treatment with DenSpm.

**FIG 7:**
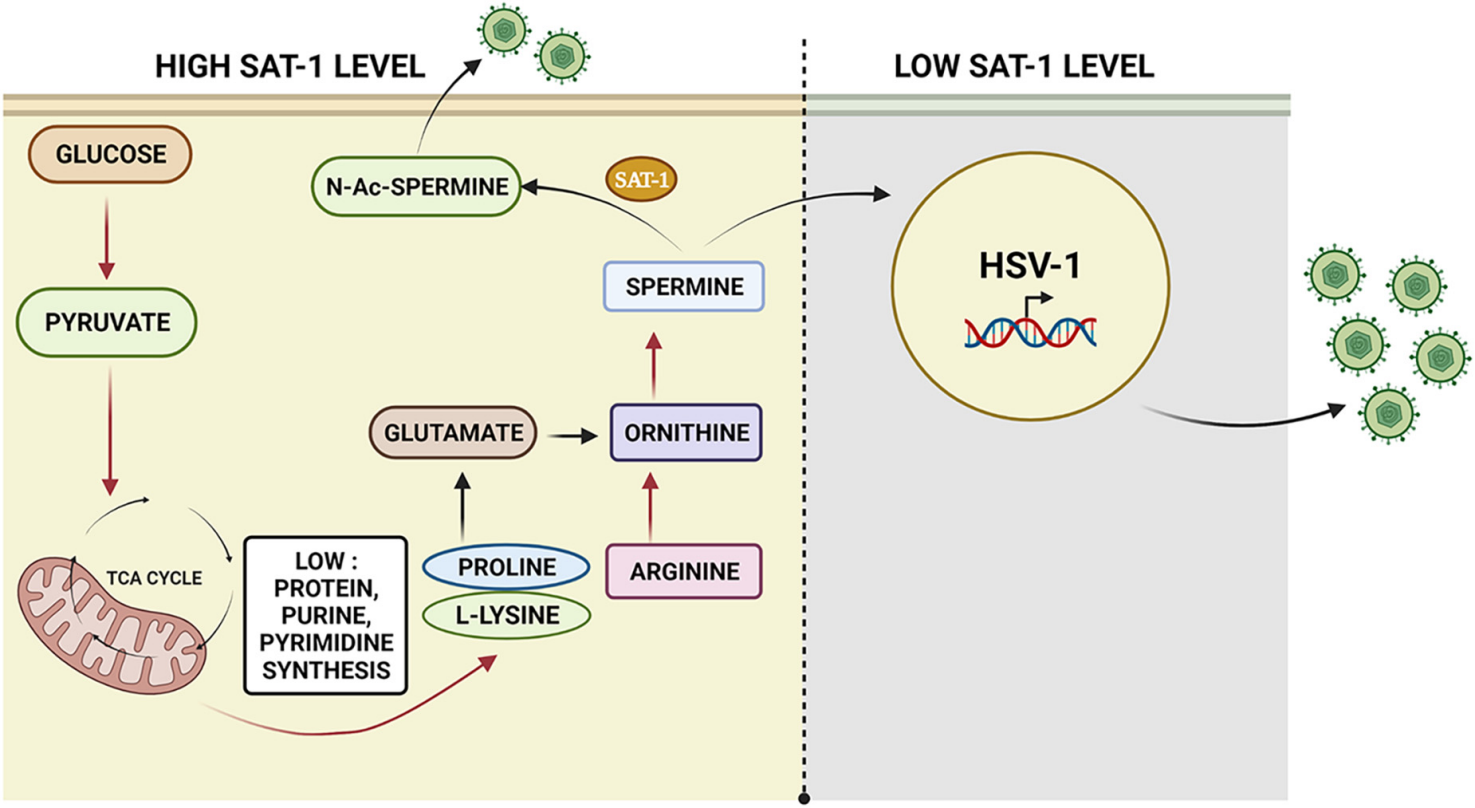
Summary of HSV-1 mediated metabolic reprogramming. A model representative of possible biochemical events during the latent HSV-1 infection. A sequential downregulation of a critical energy pathway, amino acid metabolism, arginine to spermine conversion via ornithine, and the resultant SAT-1 mediated acetylation of spermine and low protein and nucleotide synthesis creates a nonpermissive environment for virus replication and potentially could drive HSV-1 toward latency. In contrast, when host metabolic reprogramming did not influence spermine levels, HSV-1 could replicate normally. Image generated using BioRender.

Similarly, cultured dopaminergic human neurons were treated with DenSpm and infected with HSV-1 at two different MOIs. Both showed decreased levels of ICP0 proteins (Fig. 6D). Plaques assay data further showed a similar trend of HSV-1 inhibition in the presence of DenSpm at both 0.1 and 1 MOI (Fig. 6E). To check whether ocular treatment of DenSpm could reduce HSV-1 infection, we used human corneal explants. Human corneal tissue pretreated with 50 μM DenSpm inhibited HSV-1 infection (Fig. 6F), further supporting our hypothesis in a tissue model (Fig. 6G). Our overall observations are summarized in schematic form in Fig. 7.

## DISCUSSION

The typical fate of HSV-1 infection is primary infection followed by lifelong latency in the sensory neurons and trigeminal ganglia. The latent state protects the virus from commonly prescribed drugs such as acyclovir and analogs (3). Understanding the biochemical events during latent viral infection is critical for therapeutic development. Metabolomics analysis can provide unique insights into host-virus infection dynamics at different stages. HPLC coupled with tandem mass spectrometry (MS/MS) is the most common method employed for metabolomics studies (19). In recent years, metabolism-related studies have highlighted the maintenance and propagation of viruses in their hosts (9, 10, 20). However, most of these studies have been carried out using *in vitro* cell cultures. The intrinsic metabolic differences between *in vivo* and *in vitro* conditions present challenges for translating cell culture-based assays to clinical practice (12). Therefore, analysis of *in vivo* samples is a vital resource to generate more conclusive preclinical information. There is no data currently available on the metabolic state of host tissue after prolonged infection, but such information could provide targeted therapeutic directions to control the acute infection state. Metabolomics analysis is an essential tool for understanding HSV-1-induced pathogenesis.

This study analyzed TG tissue with established HSV-1 latency to understand the specific metabolic profile during latency. We found that (i) metabolite profiling could successfully identify and differentiate HSV-1-induced metabolic alterations, (ii) HSV-1 latency could be the outcome of metabolic reactions that downregulate proviral metabolic pathways and ultimately make a hostile microenvironment for virus replication, and (iii) a cellular polyamine, spermine could be essential for active viral replication. Herpesviruses encode multiple glycoproteins, playing essential roles in viral entry (4, 5). As recently shown, the synthesis of these glycoproteins crucially depends on pyrimidine biosynthesis (9). Virions induce pyrimidine biosynthesis to increase UDP-sugars, promoting the glycosylation of viral envelope protein(s). Inhibition of pyrimidine biosynthesis leads to a decrease in glycosylation of viral envelope protein, gB, which lowers human cytomegalovirus DNA accumulation levels (11). The link between pyrimidine biosynthesis and UDP-GlcNAc is crucial for replicating diverse viruses such as herpesvirus, orthomyxovirus, and rhabdovirus (11). Interestingly, UDP-GlcNAc or UDP-glucose supplementation rescues viral growth in the face of pyrimidine biosynthesis inhibition, suggesting that it could be an essential requirement for viral infections in general. As observed in this study, the downregulation of pyrimidine biosynthesis and GlcNAc could create an adverse environment for viral growth.

Our pathway analysis identified glutamate metabolism as the most highly affected pathway. Glutamate is an abundant neurotransmitter present in the ganglionic region, synthesized from its precursor, glutamine. Low glutamine levels in ganglions are associated with stress induction and impaired lymphocyte function (21). Such disturbances contribute to the development of craniofacial pain (22, 23). Facial pain along the branches of the cranial nerve could be attributed to active or inactive viral lesions that may reactivate in many immunocompromised states. Studies have shown that the level of glutamine and stress consequently impact the population of CD8^+^ T cells in trigeminal ganglions that regulate viral replication (21). The lower glutamate levels observed in our study could result from impaired lymphocyte infiltration in the infected TG microenvironment. Studies on how long immune cells respond to latent HSV-1 could shed more light on their likely contribution in an altered metabolic environment. Other affected pathways include arginine biosynthesis, arginine and proline metabolism, glycine, serine and threonine metabolism, and aminoacyl-tRNA biosynthesis. The downregulation of these pathways indicates decreased protein metabolism in mice TGs harboring latent HSV-1 infection compared to a mock-infected mice population. The decrease in amino acid concentrations shows that latency may result from low energy and nutrition-depleted conditions. Decreased protein metabolism affects the other energy-generating pathway (e.g., the TCA cycle) and may also limit the host factors required for viral protein synthesis.

To validate our data analysis and check whether depletion of marker metabolites could inhibit the viral replication in HCE cells, a natural target for HSV-1 primary infection, we targeted the polyamine synthesis pathway. Polyamines like spermine and spermidine are essential metabolites present in the host and HSV-1 (24, 25). Spermine is normally present in HSV-1 nucleocapsid while spermidine aggregates in the viral envelope. Most of the cellular polyamines are bound to the cellular DNA, RNA, proteins, and other molecular components (26). During infection, spermine facilitates encapsidation of the viral genome while depletion of polyamines leads to abnormal production of virions (27, 28). Aside from a recent report, these polyamines are poorly characterized in viral replication (29). We found that a spermine analog, DenSpm, effectively increases the expression of SAT-1. With low RFP expression in imaging data, low spermine conditions influence the late viral gene expression. Supporting data not presented in the current study indicates that HSV-1 can complete the expression of early genes but not late genes due to low glucose, purine, pyrimidines, and polyamine levels in depleted spermine conditions. In addition to the murine TG, human neurons and human corneal infection models showed consistent efficacy in regulating HSV-1 infection when treated with DenSpm. In the future, it would be interesting to study whether increasing these metabolites favor a lytic state over a latent state. It is essential to note the relationship of our findings to the existing correlation between the reactivation of HSV-1 and host stress. A transient increase in brain polyamine metabolism is a common reaction to physical, mental, and hormonal external stress stimuli (30). This temporary upregulation of spermine in neuronal cells could be enough to trigger the viral reactivation cycle. An available example from the literature shows that polyamines could be the new targets for potential therapeutic interventions. For instance, blocking polyamine synthesis with Difluoromethylornithine (DFMO), which inhibits putrescine, a compound of polyamine synthesis pathway, upregulates neuronal α4β2 and α7 surface levels and promotes nicotine-mediated neuroprotection (31).

HSV-1 latency-reactivation cycle is a result of complex virus-host interactions. It is not very clear whether the metabolic changes during latency are driven by the host response to infection, HSV itself, or both. Apart from low levels of a few viral proteins, a virally encoded transcript called latency-associated transcript (LAT) is abundantly expressed during latency (14). LAT has many known regulatory functions. It encodes several micro-RNAs and noncoding RNAs, which regulate local gene expression and host signaling pathways, which in turn, contribute to the latency-reactivation cycle. Because LAT was the major viral transcript expressed in latently infected neurons, future studies using LAT null mutant could shed more light on LAT-dependent/independent metabolic changes in neuronal tissues. Likewise, studies are needed to identify the metabolic changes that occur during reactivation from latency. It remains to be determined whether metabolic conditions become more favorable when reactivation occurs *in vivo*. Furthermore, understanding whether virus-induced host metabolic reprogramming is shared among evolutionarily diverse viral families may provide novel avenues for broad-spectrum antiviral therapeutic intervention.

## MATERIALS AND METHODS

### Cells and viruses.

The human corneal epithelial (HCE) cell line (RCB1834 HCE-T) was procured from Kozaburo Hayashi (National Eye Institute, Bethesda, MD). The HCE cells were cultured in minimum essential medium (MEM) (Life Technologies, Carlsbad, CA) supplemented with 10% fetal bovine serum (FBS; Life Technologies) and 1% penicillin/streptomycin (Life Technologies). The KOS strain and GFP expressing HSV-1 strains were obtained from Patricia G. Spear (Northwestern University, Chicago, IL) and Dr. Prashant Desai (Johns Hopkins University). Paul Kinchington (University of Pittsburgh) provided the dual-tagged KOS strain of HSV-1 (RFP driven by gC promoter and GFP driven by ICP0 promoter). HSV-1 McKrae strain was used for mouse and TG experiments. Virus stocks were prepared and titrated in Vero cells and stored at −80°C until used. Repeated freeze-thaw of the virus aliquots was avoided. A neuronal cell line, Lund human mesencephalic (LUHMES), was obtained from Dr. David Bloom (University of Florida) and cultured as per the detailed methods outlined (32). Briefly, poly-l-ornithine and fibronectin-coated tissue culture flasks and plates were used to maintain the cells. Proliferation medium (DMEM:F12 [ATCC]) containing 1% N2 supplement (ThermoFisher), 1× penicillin-streptomycin-glutamine solution (ThermoFisher), and recombinant human FGF-basic (fibroblast growth factor; Peprotech) was used to grow the cells. Once 50% confluence was achieved, cells were differentiated in a differentiation medium with 1 μg/mL tetracycline hydrochloride (Sigma), 1 mM N6,2′-O-dibutyryladenosine 3′,5′-cyclic monophosphate sodium salt (Sigma), and 2 ng/mL glial cell-derived neurotrophic factor (GDNF). At 3 days after differentiation, cells were treated for 48 h with DenSpm containing differentiation medium. On the 5th day after differentiation, cells were used for infection assays. Samples were collected for Western blot and plaque assay.

### Infection of murine corneas.

C57BL/6 mice were used for *in vivo* studies. All animal care procedures were performed following institutional and NIH guidelines, with prior approval from the Animal Care Committee at the University of Illinois at Chicago (ACC protocol 17-077). A total number of 10 C57BL/6J mice were divided into two groups. Mice from both groups were anesthetized to scarify their cornea in a 3 × 3 grid using a 30-gauge needle. Corneas designated to the infected group were infected with HSV-1 McKrae (1 × 10^5^ PFU). The control group was infected with mock treatments. Mature virus production in ocular wash samples was assessed using plaque assays collected on 2, 4, 7, 14, and 21 days postinfection (dpi). The animals were euthanized 30 days after infection, and trigeminal ganglion tissue was collected and snap-frozen in liquid nitrogen.

### Antibodies and chemical reagents.

HSV-1 gB (ab6506) and Anti-HSV1 ICP0 antibody (5H7; ab6513) were purchased from Abcam (Cambridge, United Kingdom). SAT-1 (D1T7M) and GAPDH (10494-1-AP) were purchased from Cell Signaling Technology (Danvers, MA). N1,N11-diethylnorspermine was purchased from Santa Cruz Biotechnology (catalog number SC-204114), and difluoromethylornithine was obtained from Selleckchem (catalog number S4582).

### Metabolite extraction.

Freshly collected tissue was snap-frozen in liquid nitrogen and stored at −80°C until analysis. For extraction of metabolites from frozen tissue, 80% (vol/vol) of cold methanol was added to tissue at a concentration of 20 μL/mg tissue and homogenized with an ultrasonicator. Next, 80% (vol/vol) methanol was used to clean the probe between samples to avoid cross-contamination. Then, 200 μL of homogenate was transferred into a tube preadded with 800 μL of 80% (vol/vol) methanol (cooled to −80°C) and incubated at −80°C for at least 4 h. The samples were vortexed vigorously for 1 min and then centrifuged at 20,000 × *g* for 15 min at 4°C. The extract containing metabolite was stored at −80°C until transferred to the core. The protein pellet was quantified using the bicinchoninic acid assay (BCA assay). The extracted solution was dried using SpeedVac and reconstituted using 50% acetonitrile followed by vortexing for 30 s. This was followed by centrifugation for 15 min at 20,000 × *g*, 4°C, and the supernatant was collected for LC-MS analysis.

### Method for hydrophilic metabolites profiling.

All samples were analyzed by high-performance liquid chromatography (HPLC) and high-resolution mass spectrometry with tandem mass spectrometry (HPLC-HRMS/MS). Specifically, the system consisted of a Thermo Q-Exactive in line with an electrospray source and an Ultimate3000 (Thermo) series HPLC consisting of a binary pump, degasser, and auto-sampler outfitted with an Xbridge Amide column (Waters; dimensions of 4.6 mm × 100 mm and a 3.5 μm particle size). The mobile phase A contained 95% (vol/vol) water, 5% (vol/vol) acetonitrile, 20 mM ammonium hydroxide, 20 mM ammonium acetate, pH = 9.0; B was 100% Acetonitrile. The gradient was as follows: 0 min, 15% A; 2.5 min, 30% A; 7 min, 43% A; 16 min, 62% A; 16.1 to 18 min, 75% A;18 to 25 min, 15% A with a flow rate of 400 μL/min. The capillary of the ESI source was set to 275°C, with sheath gas at 45 arbitrary units, auxiliary gas at 5 arbitrary units, and the spray voltage at 4.0 kV. In positive/negative polarity switching mode, an *m/z* scan range from 70 to 850 was chosen and MS1 data were collected at a resolution of 70,000. The automatic gain control (AGC) target was set at 1 × 10^6^ and the maximum injection time was 200 ms. The top 5 precursor ions were subsequently fragmented, in a data-dependent manner, using the higher energy collisional dissociation (HCD) cell set to 30% normalized collision energy in MS2 at a resolution power of 17,500. Data acquisition and analysis were carried out by Xcalibur 4.1 software and Tracefinder 4.1 software, respectively (both from Thermo Fisher Scientific).

### Data processing.

All statistical analyses followed recommended procedures as described in previously published protocols (10). A *P* value less than 0.05 was used as the cutoff for statistical significance. The MetaboAnalyst software (https://www.metaboanalyst.ca) was applied for metabolomics analyses (10). If metabolites were found to be >30% missing values, they were removed from calculations. Otherwise, missing values of one metabolite were replaced by a value of half of the minimum positive value of the same metabolite in the original data set. Before statistical analysis and pathway analysis, the peak area was normalized by dividing each reported peak area by the sample-specific normalization factor using log transformation and Pareto-scaling to achieve a Gaussian distribution, as described and recommended (10). To find statistically significant differences between sample groups (WTC versus WTI), unsupervised principal component analysis (PCA) and supervised orthogonal partial least square-discriminant analysis (OPLS-DA) were performed. Permutation testing (N = 100), Q2 (>0.5), and R2Y (>0.5) were used as quality parameters for model validation. Metabolomics data have been deposited to the European molecular biology laboratory’s European bioinformatics institute (EMBL-EBI) MetaboLights database (11) with the identifier MTBLS2509. The complete data set can be accessed at https://www.ebi.ac.uk/metabolights/MTBLS2509.

### Spermine detection and quantitation.

The cells from the experimental condition were harvested in Hanks’s buffer (Life Technologies Corporation, Grand Island, NY) and the samples were centrifuged for 5 min at 6,000 rpm, 4°C. The supernatant was discarded, and the pellets were washed with PBS and centrifuged for 5 min at 6,000 rpm at 4°C. The pellets were treated with 250 μL of 2% (vol/vol) perchloric acid (Sigma-Aldrich, St. Louis, MO) in the dark. They were sonicated and stored at 4°C for 24 h. After the incubation, the cell homogenate was centrifuged at 11,500 × *g* for 30 min at 4°C. Next, dansylation of the samples was performed by adding 400 μL of freshly prepared dansyl chloride (Sigma-Aldrich, St. Louis, MO) in acetone (Fisher Scientific, Geel, BE) to 200 μL of the collected supernatants. The samples were vortexed for 15 s and 200 μL of supersaturated sodium carbonate was added to them. Then the samples were incubated for 16 h at room temperature in the dark. Excess dansyl chloride was neutralized by the addition of 100 μL of l-proline (Sigma-Aldrich, St. Louis, MO) solution in water. The samples were incubated in the dark for 30 min and 500 μL toluene was added. The samples were agitated for 1 min and then centrifuged for 10 min at 4350 × *g* at 4°C. Toluene supernatant phase was collected and 10 μL of each was added in 2.00 cm increments from the bottom edge of the TLC plate (TLC silica gel 60 F_254_, Sigma-Aldrich, Burlington, MA). The plate was then placed in a presaturated chromatographic chamber with a mobile phase of cyclohexane (Sigma-Aldrich, St. Louis, MO):ethyl acetate (Mallinckrodt Inc., St. Louis, MO) (3:2, vol/vol) for 20 min. The plate was air dried in the dark at room temperature and scanned for spermine bands under a UV irradiation chamber. After detection, the zone containing dansylated spermine was scrapped off from the TLC plate and dissolved in 200 μL of toluene, and vortexed for 15 s. The supernatant was collected, and fluorescence was measured (Ex 365, Em 505) with the BioTek Synergy H1 microplate reader. The fluorescence recorded for each spot was then compared with known series of concentration curve readings generated for spermine.

### Cell viability assay.

HCE cells were used to measure the percent viability of cells at different concentrations of DenSpm. A 96-well plate of HCE cells was seeded and incubated overnight before treatment with DenSpm at various concentrations starting from 250 μM. DMSO (at vehicle concentration) was used as a control. After 72 h of treatment, the cell viability was recorded using the MTT assay. In brief, 50 μL of MTT reagent (5 mg/mL) dissolved in PBS, was added to each reaction well and incubated at 37°C in the dark. After 3 h, formazan crystals were dissolved in acidified isopropanol by shaking the plate at 25 rpm for 15 min in the dark. The supernatant was transferred to a new 96-well plate. The optical density of the resultant mixture was measured at 550 nm while the reference wavelength was 630 nm. The percent viability was calculated.

### Western blot.

For *in vitro* experiments, the protein expression was evaluated by Western blot. In brief, the cells were dissociated using Hanks’ cell dissociation buffer, while the protein was isolated by radioimmunoprecipitation assay (RIPA) buffer (Sigma-Aldrich, St. Louis, MO). The protein samples were mixed with LDS sample loading buffer (35%) and β-mercaptoethanol (5%) (Bio-Rad, Hercules, CA). The resultant mixture was heated at 95°C for 10 min followed by an electrophoretic run on Invitrogen Mini Gel Tank (Fisher Scientific) through precast gels (4 to 12%). The proteins from the gel were transferred to a nitrocellulose membrane (Fisher Scientific) using iBlot (Invitrogen). The membranes were further exposed to 5% milk/tris-buffered saline with tween 20 (TBS-T) for 1 h to block nonspecific sites on a membrane, followed by overnight incubation with respective primary antibodies at 4°C on a shaker. On the next day, the unbound antibodies were washed with TBS-T and the membrane was incubated with secondary antibody conjugated with horseradish peroxidase (HRP) for 1h at room temperature. Further, after washing with TBS-T, the membrane was exposed to SuperSignal West PICO plus chemiluminescent substrate (34577) and or SuperSignal West Femto maximum sensitivity substrate. Image-Quant LAS 4000 biomolecular imager (GE Healthcare Life Sciences, Pittsburgh, PA) was used for the visualization of protein bands.

### Quantitative real-time PCR.

RNA expression was analyzed by quantitative real-time PCR (qRT-PCR). The RNA was isolated using TRIzol (Life Technologies). The isolated RNA was converted to cDNA (2 μM) using a High-Capacity cDNA Reverse Transcription kit (Applied Biosystems Foster City, CA). Further, the cDNA was mixed with Fast SYBR Green Master Mix (Life Technologies) and qRT-PCR was performed using QuantStudio 7 Flex system (InvitrogenTM Life Technologies).

### Plaque assay.

HSV-1 infected HCE cells were centrifuged at 3000 rpm for 5 min after dissociation with Hanks’ buffer. The cell pellets were then suspended in 1 mL Opti-MEM (Thermo Fisher Scientific). The cells were sonicated at 30% amplitude for 15 s to release intracellular infectious viral particles. The cell lysate was diluted using Opti-MEM and overlaid on a monolayer of Vero cells. After 2 h of infection, the Opti-MEM was replaced by complete DMEM (10% FBS and 1% PenStrap) containing 0.5% wt/vol methylcellulose (Fisher Scientific). The culture plate was further incubated at 37°C with 5% CO_2_ for 72 h. The resultant plaques were visualized by fixing the cells with 100% methanol and staining the cells with crystal violet solution. The plaques were counted visually and multiplied with respective dilution factors to obtain plaque forming units/mL.

### SAT1 short interfering RNA transfection.

A Dicer-Substrate Short Interfering RNAs (DsiRNAs) TriFECTa kit (Integrated DNA Technologies) with predesigned SAT1-specific short interfering RNA (siRNA) molecules were used for transfections in this study. Cells grown in a 6-well plate at 45% confluence were transfected for 60 h using 5 nM SAT1 siRNA molecules. At 8 h posttransfection, Opti-MEM was replaced with DMEM medium at the same time DenSpm was added to the treatment group. Appropriate controls were prepared and tested for transfection and knockdown efficacy as per the manufacturer’s recommendations.

### HSV-1 infection of murine trigeminal ganglion explants.

Murine TGs were collected from C57BL/6 mice (*n* = 9). Trigeminal ganglions were divided into three groups and cultured *ex vivo* in MEM medium supplemented with 5% antibiotic (Thermo Fisher Scientific) and 10% FBS (Sigma-Aldrich) at 37°C, with 5% CO_2_ for 24 h. Each pair of cultured TGs were pretreated separately with DenSpm (50 μM) or Acyclovir (50 μM) in a complete medium. After 24 h of pretreatment, each pair of TGs were incubated in 100 μL of HSV-1 McKrae (1 × 10^5^ PFU) in an Opti-MEM medium (Thermo Fisher) for 2 h. The TGs were gently transferred back to treatment wells for another 24 h. The assay was terminated at 48 hpi, and the 100 μL culture supernatant was processed for plaque assay to evaluate the antiviral ability of DenSpm in cultured TGs. Untreated TGs served as a control.

### Human corneal model of HSV-1 infection.

Human corneas were obtained from fresh cadavers (age range, 17 to 88 years) supplied by the Illinois (Chicago) and Midwest Eye Banks (Ann Arbor). The corneas were cultured *ex vivo* in MEM medium supplemented with 5% antibiotic-antimycotic (Thermo Fisher Scientific) and 1% insulin-transferrin-sodium-selenite (Sigma-Aldrich) at 37°C, with 5% CO2. The cultured corneas were pretreated with DenSpm (50 mM) in a complete medium. After 24 h of pretreatment, the corneas were gently scarified with a sterile 30-gauge needle, and infected with 10^7^ PFU of HSV-1 (KOS-Dual) in Opti-MEM medium (Thermo Fisher Medium) containing DenSpm (50 mM). At 24 h postinfection, the culture medium was replaced by a complete medium containing DenSpm (10 mM). The assay was terminated at 48 hpi, and the culture supernatant was processed for plaque assays to evaluate the antiviral ability of DenSpm in cultured human corneas. Uninfected corneas served as a control.

### Statistical analysis.

Each experiment was carried out in triplicates unless specified, the respective error bars in all figures represent SEM. Unpaired Student's *t* test was used to compare the data between the two groups. The *p* was considered significant when *P* = 0.05. The *P* values indicated in the figures are (*, *P* < 0.05; **, *P* < 0.01; ***, *P* < 0.001; ****, *P* < 0.0001).
